# Circulating Human Papillomavirus DNA in Head and Neck Squamous Cell Carcinoma: Possible Applications and Future Directions

**DOI:** 10.3390/cancers14235946

**Published:** 2022-12-01

**Authors:** Dauren Adilbay, Saudamini Lele, John Pang, Ameya Asarkar, Jason Calligas, Cherie-Ann Nathan

**Affiliations:** 1 Department of Otolaryngology/Head Neck Surgery, LSU Health, Shreveport, LA 71101, USA; 2 Surgical Service, Otolaryngology Section, Overton Brooks VA Medical Center, Shreveport, LA 71101, USA

**Keywords:** HPV, oropharyngeal cancer, ctDNA, squamous cell carcinoma, p16, liquid biopsy

## Abstract

**Simple Summary:**

Circulating human papillomavirus DNA is a promising tool in the management of HPV-positive oropharyngeal cancer. The potential use of this tool will substantially change the way we treat, diagnose, and follow-up these patients.

**Abstract:**

There has been a rising trend in HPV-induced head and neck cancers in the last several decades. This subgroup of squamous cell carcinoma is mostly located in the oropharynx and comprises a subset of patients who are typically younger and without the usual risk factors of smoking and alcohol use. As the prognosis of HPV-induced OPC is more favorable, there is a desire to properly select these patients for de-intensification protocols while identifying individuals who may suffer treatment failure. Here, we describe recent developments in circulating tumor HPV DNA as a marker of HPV-positive oropharyngeal cancer that can potentially be used as a diagnostic tool to stratify patients for de-escalation strategies and to survey for recurrence.

## 1. Introduction

Human papillomavirus (HPV)-induced oropharyngeal cancer (OPC) is a distinct entity with a rising incidence in the US and across the world [1]. This subset of head and neck cancers has a better response to treatment and prognosis than non-HPV cancers [2]. Current treatment paradigms are shaped by a desire to diagnose patients at an earlier stage, select patients for de-intensification protocols (which is being explored in many clinical trials) [3,4,5], and identify patients with a poorer prognosis who are subject to recurrence. Such aims are predicated on the identification and validation of biomarkers for HPV-induced oropharyngeal cancer (HPV-OPC).

Circulating cell-free tumor DNA, also called liquid biopsy, is being used in several fields and can serve as a potential marker in cancers attributed to viruses [6,7]. Several trials were successfully conducted in nasopharyngeal carcinoma, for which circulating Epstein–Barr virus DNA was found to be an effective biomarker [8,9]. Similarly, circulating tumor HPV DNA (ctHPV DNA) is a potential biomarker for OPC cases attributed to HPV, and it is also being investigated to select patients for de-escalation therapy [10]. Furthermore, ctHPV DNA may be used in surveillance and may have a comparable or higher specificity relative to current surveillance modalities. In this review, we discuss the current state and ongoing research using ctHPV DNA as a marker with different applications in the management of HPV-OPC.

## 2. Circulating Tumor DNA (ctNDA)

Circulating cell-free DNA (cfDNA) has been utilized in several fields of medicine since its advent in the mid-twentieth century. Cell-free DNA is fragmented DNA with an average size of 160–200 bp, and it is released into the blood and other body fluids through the apoptosis or necrosis of cells [11]. It has a short half-life ranging from 16 min to a few hours with an average blood concentration of 10–30 ng/mL in healthy adults. The cfDNA that has been released from tumor cells is referred to as circulating tumor DNA (ctDNA). CtDNA can be distinguished from cfDNA by the presence of specific somatic mutations. Various studies have demonstrated increased levels of ctDNA in patients with cancer, and recent studies have also identified specific epigenetic and genetic characteristics of head and neck cancers [7,12,13,14,15,16,17]. However, its consistent utility in the clinical setting is yet to be explored.

## 3. Circulating HPV DNA Detection Methods and Available Diagnostic Tests

The most common target for detecting ctDNA for HPV-OPC is the E6 or E7 open reading frame [18]. One area of caution is that the classic paradigm of HPV oncogenesis, which involves the insertion of HPV into the human genome (i.e., integration) and the subsequent coaptation of host cellular machinery for the overexpression of E6 and E7, may not apply to all HPV-mediated tumors. The next-generation sequencing of HPV-OPC revealed a subset with HPV-only sequences without the presence of HPV-human DNA [19]. This is evidence for non-integrated forms of HPV-OPC. In addition, there is a second subset of HPV-OPC that under-expresses E6 and E7 while overexpressing oncoproteins E2, E4, and E5 [20]. In conclusion, the ideal so-called target (s) for ctDNA detection methods is an evolving area of discussion and requires continued investigation.

In addition to target variation, three different methods of assaying for ctDNA have been employed to date, most commonly quantitative PCR (qPCR), next-generation sequencing (NGS), and the more novel digital droplet PCR (ddPCR) [18,21]. Although a detailed description of each technique is outside the scope of this review, quantitative PCR involves the creation of a pre-selected array of DNA probe pairs and the creation of standard curves for each probe set so the amount of DNA of interest can be quantified based on the relative intensity of fluorescence. Although multiplex systems have reduced the necessary labor, qPCR can be labor-intensive and may not be best-suited to scenarios with a limited sample availability. Next-generation sequencing involves using a DNA primer to directly determine the nucleotide sequence of 150–200 base pair segments of DNA. However, designing primers specific to the region of interest is challenging due to specificity and thermodynamic restrictions that can be challenges for both PCR and NGS. Moreover, only 150–200 base pair reads are typically possible. Finally, digital droplet PCR involves the emulsification of a reaction mix into thousands of nanoliter-sized droplets, such that an absolute number of DNA copies can be determined without the need for standard curves. Early studies suggested that ddPCR and NGS may have better accuracy than qPCR [22].

## 4. The Use of ctHPV DNA as a Diagnostic Tool and Cancer Screening

An earlier stage at diagnosis correlates with improved survival in HPV-related oropharynx squamous cell carcinoma [23,24]. In the initial validation cohort of the paradigm that became the American Joint Commission on Cancer (AJCC) version 8 TNM staging system, stage I exhibited an 88% 5-year survival rate compared with stage III patients who exhibited a 65% 5-year survival rate [23]. Rettig et al. demonstrated that circulating tumor HPV DNA could be detected well in advance of cancer diagnosis in some but not all patients (range of 19–43 months), illustrating that the earlier diagnosis of HPV-OPC is possible and the treatment of a more localized disease may bear therapeutic advantages [25].

The current gold standard for the diagnosis of HPV-OPC is tissue diagnosis on either the biopsy of the primary site or the fine needle aspiration of a metastatic lymph node, which is not without limitations. Clinical facilities in low-resource settings may not be well-suited to obtain, store, and process specimens for off-site histologic diagnosis. In addition, fine needle aspiration may yield insufficient cells for p16-immunohistochemistry, so additional biopsies may be necessary to obtain more tissue for determining p16 status. P16 status is critical to diagnostic work-up as it bears a strong prognostic indicator related to HPV-16 status [26]. In this regard, liquid biopsy techniques may be a useful adjunct to traditional diagnostic pathways in that they can expedite diagnosis when referral times to an otolaryngologist are lengthy or when patient comorbidities either preclude general anesthesia or require additional cardiopulmonary clearance for general anesthesia. Complications of operative laryngoscopy, while rare, include anesthetic complications, medication errors, dental injuries, and esophageal perforations. Moreover, tissue biopsy does not guarantee an accurate diagnosis, as false-negative results can occur in a setting of low-volume or submucosal disease due to sampling errors.

To date, the detection of ctDNA in HPV-OPC has been predicated on detecting circulating HPV DNA. This premise assumes that circulating HPV DNA is never observed in patients with either (1) Pnon-invasive HPV-associated lesions or (2) HPV infection without carcinogenesis. Indeed, Jeannot et al. detected no circulating HPV DNA in 18 patients with cervical intraepithelial neoplasia [27]. However, this was a small trial, and it remains possible that patients with an HPV infection but without a tumor may shed HPV DNA in plasma, though at a level below the limit of detection of current methods. A list of studies researching the sensitivity and specificity of ctHPV DNA is given in Table 1, and a detailed discussion of the results is given in the following paragraphs.

### 4.1. Sensitivity

For diagnostic purposes, sensitivity is a measure of a screening tool’s efficacy. A high sensitivity ensures that few, if any, patients with HPV-OPC would test negative for ctDNA. For patients with a high pre-test probability of HPV-OPC in whom a false-negative ctDNA result is suspected, traditional tissue biopsy has a vital role.

Siravegna et al. utilized ddPCR assays for the HPV E7 oncoprotein in HPV 16, 18, 33, 35, and 45 in 70 cases of HPV-OPC and 70 controls (HPV-negative (45) and non-cancer (25)) [28]. With only one false-negative finding, they reported a sensitivity of 98.4%. Mattox et al. detected ctDNA via the ddPCR, NGS, and qPCR targeting of E6 in 66 patients with HPV16-related OPC and found the sensitivity of NGS to be 75% vs. 8.3% (ddPCR) and 2.1% (qPCR). [22] Veyer et al. utilized ddPCR targeted to HPV E6 and identified HPV16 ctDNA in 47 of 66 patients, demonstrating a sensitivity of 71% [29]. Tanaka et al. utilized ddPCR to target HPV E6 and E7, and they reported a sensitivity of 93% (39/42) for HPV-OPC and 100% (6/6) for patients with unknown primary [30]. Ahn et al. performed qPCR using probes for HPV16 E6 and E7 on 93 patients and found a sensitivity of 67% [8]. Hanna et al. utilized ddPCR targeting E7 in the plasma of patients, and they found a sensitivity of 76% in 21 patients—all with metastatic disease who were previously treated [31]. Chera et al. analyzed 103 patients whose plasma was tested for ctDNA using ddPCR for HPV16 E7 and E7 from multiple HPV strains—the sensitivity was reported to be 89% [32]. Cao et al. utilized a combination of conventional PCR for L1, E6, and E7 and qPCR for E6 and E7 in 40 patients [33], and they detected ctDNA in 65% of patients. Wang et al. detected plasma ctDNA in 91% of patients with HPV-OPC, although the sample size was only 22 patients [34]. They utilized ddPCR targeting E7 in HPV16 and 18, as well as mutations in oncogenes including TP53, PIK3CA, and others. Rettig et al. utilized ddPCR for HPV16 sequences (unspecified) in seven patients and reported a sensitivity of 43% [25].

In a small study of 17 patients with HPV-related carcinoma of the cervix, Leung et al. tested the utility of a novel detection method called HPVseq, which utilizes 150 bp long “bait” probes to detect E6 and E7, as well as full-length HPV 16, 18, 33, 45, 31, 33, and 35. They reported a 100% sensitivity, as all patients had detectable ctDNA down to 0.03 copies/mL [35].

### 4.2. Specificity

No treatment modality for head and neck cancer is free of adverse effects. Surgery, radiation, and cytotoxic chemotherapeutics all bear permanent side effects such that the treatment of false positives is impermissible. Consequently, it is critical that diagnostic pathways incorporating ctDNA never produce a false positive.

Ahn et al. performed qPCR using probes for HPV16 E6 and E7 and found a specificity of 100% in nine patients with HPV-negative tumors [8]. Rettig et al. utilized ddPCR for HPV16 sequences (unspecified) and identified a specificity of 100%, as zero of the 100 matched controls tested positive [25]. Tanaka et al. utilized ddPCR to target HPV E6 and E7, and in their control group of patients with HPV-unrelated cancers, they reported a specificity of 97% (31/32) for HPV-OPC and a specificity 100% (2/2) for patients with unknown primary [30]. Tewari et al. utilized traditional PCR to test for ctDNA in 407 controls, and they found that zero tested positive [36]. However, their report was scrutinized for possibly having faulty assay techniques and sample degradation [21]. In the comparison control cohort of Siravegna et al., one of 70 controls was a false positive for ctDNA according to ddPCR assays for HPV E7, yielding a specificity of 98.6% [28]. Chera et al. analyzed 115 controls and reported a specificity rate of 97% [32].

### 4.3. Considerations of ctDNA for Diagnosis

Most methods for detecting ctDNA in HPV-OPC demonstrate sensitivity rates of 70–80%, although sensitivity can be improved depending on the used PCR technique. Increasing the number of targets (including E2, E4, and E5) would likely increase sensitivity rates and diagnostic utility. However, increasing the number of targets may also introduce the added possibility of false positives and thereby decrease the specificity rate. As this area continues to expand, distinct screening and confirmation probe sets may be necessary.

## 5. Circulating HPV DNA in Treatment Stratification, Cancer Surveillance, and Prognostics 

Currently, surveillance is typically predicated on physical exams at regular intervals and post-treatment PET–CT scans. Unfortunately, even with state-of-the-art treatment, recurrence is not uncommon, reaching 26% (9–26%) in 5 years and 36% in 8 years [37,38,39]. Circulating HPV DNA detection may be a method that helps prognosticate or supplement current recurrence detection methods. CtHPV DNA is being investigated in three major areas:

1. Pretreatment levels of circulating HPV DNA as a prognostic tool.
2. As an early recurrence detection tool for follow-up after definitive treatment.
3. As a tool to define the treatment strategy and implementation of deintensification therapies.

### 5.1. Pretreatment Levels of HPV DNA as a Prognostic Tool

Although HPV-positivity is associated with a favorable prognosis, up to 35% of patients develop an aggressive disease [39]. Currently, there is interest in identifying pre-treatment biomarkers to predict aggressive subsets of HPV-positive diseases.

Emerging data suggest that ctHPV DNA bears promise as a prognostic tool; however, the timing of the ctDNA assessment and assay technique are critical. In a recent study by Cao et al. where HPV-OPC patients were all of the same stage (AJCC Stage III), both pretreatment ctDNA values and ctDNA two weeks into chemoradiation demonstrated prognostic value [40]. Higher levels of pre-treatment ctHPV DNA correlated with a higher risk of progression (HR = 1.06 (95%CI of 1.01–1.12) per 1000 copies ctDNA/mL; *p* < 0.03). Furthermore, there was a significantly lower risk of tumor progression in patients with a significant increase in ctDNA two weeks into chemoradiation (HR = 0.11 (95%CI of 0.01–0.95; *p* < 0.05). Notably, smoking status did not predict freedom from persistence in this cohort.

The timing of ctDNA measurement is important, as Dahlstrom et al. did not observe that patients without detectable pre-treatment ctDNA had a more favorable disease course than patients with detected ctDNA [41]. One major difference between this and other studies is that Dahlstrom et al. did not measure ctDNA in the initial phase of chemoradiation. The differences in findings may also be attributed to the assay technique, as the authors performed qPCR for E6 and E7 regions while Cao et al. utilized ddPCR.

The importance of the assay technique was also highlighted in a study by Veyer et al. on 66 patients with HPV-OPC [29]. Pre-treatment ctDNA was collected and assayed using ddPCR, and 71% of patients had positive pre-treatment ctDNA. All recurrences after chemoradiation were observed in patients with detectable pre-treatment ctDNA, and none were detected in patients without detectable pre-treatment ctDNA.

In summary, higher levels of detectable ctDNA are associated with a worse prognosis. This finding appears most convincing when ddPCR is utilized instead of qPCR. As such, despite challenges, pre-treatment levels can potentially be considered prognostic for cases with classic HPV and oropharyngeal cancer with no or minimal other concurrent risk factors.

### 5.2. As an Early Recurrence Detection Tool for Follow-Up after Definitive Treatment

Unlike HPV-negative head and neck cancer, HPV-OPC patients with recurrence can achieve high survival rates with salvage therapy. Surgery for locoregional recurrence has been shown to have two-year overall survival rate approaching 80%, and re-irradiation with either IMRT or protons can lead to a two-year overall survival rate of up to 70% [42]. Incorporating ctDNA assessment into post-treatment surveillance pathways may be beneficial if recurrences can be detected earlier, before metastatic disease.

Several studies have shown that detectable post-treatment ctDNA indicates recurrence and may precede imaging or exam findings (Table 2). Cao et al. used RT-PCR for E6 and E7 to assay for ctDNA in their cohort of 40 HPV-OPC patients. Sixty-eight percent of patients had detectable ctDNA prior to treatment [33]. Of the original 40 patients, a subset of 14 patients also had serum during chemoradiation available for analysis. Four of the 14 recurred, and in one patient with distant metastasis to the lung, HPV DNA was 111 copies/mL four months prior to the detection of the lung metastasis by imaging, at which point the HPV DNA was 542 copies/mL. The authors did not detect a difference in ctDNA levels between the four patients who recurred and the ten who did not, although this may be attributable to the assay technique, as they utilized RT-PCR instead of ddPCR.

Rutkowski et al. explored the usage of post-treatment ctHPV DNA in 66 patients with HPV-OPC. An incomplete response (i.e., disease persistence) was associated with detectable ctDNA 12 weeks after treatment, as ctDNA was detected in 28% of patients with persistence and only in 4% of patients with a complete response. No patients showed negative ctDNA developed recurrence.

Chera et al. studied 115 HPV-OPC patients treated with definitive CRT [47], and they utilized ddPCR both before and after chemoradiation with blood collection performed every 6–9 months. Two consecutive positive ctDNA tests were observed in 16 patients, 15 of whom developed recurrences. They found a positive predictive value of two-consecutive positive ctDNA tests of 94%, and the median time prior to recurrence on biopsy was 4 months.

Recently, the largest study of ctDNA for the detection of the occult occurrence of HPV-OPC was conducted by Berger et al. Digital droplet PCR was utilized as the assay technique. Of 1076 patients, post-treatment ctDNA was detected in 80 patients (7.4%). Only 2 of 80 patients with detectable ctDNA had no evidence of disease on the last follow-up. The remaining 98% either had recurrence at first follow-up or were subsequently found to have a biopsy-proven recurrence.

In summary, the absence of detectable ctDNA after treatment for HPV-OPC portends a low risk of recurrence, and depending on the technique used, a positive assay for ctDNA appears to be associated with a more aggressive course. Larger prospective studies are needed to shed light on cases with several risk factors, potential HPV-negative second primaries, and the possibility of early intervention solely based on ctHPV DNA levels. In fact, several large prospective clinical trials are recruiting patients to answer these questions, with the targeted recruitment of several thousand participants, and results are expected in 2024–2025 (NCT03942380, NCT04354064).

### 5.3. As a Tool to Define Treatment Strategy and Implementation of Deintensification Therapies

Several different strategies to de-escalate treatment are being studied in dozens of trials throughout the world. Such trials are attempting to reduce definitive radiotherapy dose, perform upfront surgery with modified adjuvant treatment, and/or use immunotherapy. Many of them face the challenge of selecting an appropriate patient population that will benefit from de-intensification while preserving oncologic outcomes. Potentially, ctHPV DNA can become such a marker. There are several ongoing clinical trials underway. The NCT05307939 trial being conducted at the Memorial Sloan Kettering Cancer Center uses ctHPV DNA levels to stratify patients for either observation or adjuvant radiation after surgery. If ctDNA is undetectable after transoral robotic surgery, the patient is stratified to a close observation group without any adjuvant therapy. The SIRS 2.0 trial uses a similar design but stratifies patients with undetectable levels of ctDNA into low and intermediate-risk groups based on final pathology (NCT05419089). The low-risk group receives close follow-up, and the intermediate-risk group receives de-intensified adjuvant RT. Investigators from the ReACT (NCT04900623) study set 40–50 Gy for the low-risk group and 50–60 Gy for the intermediate-risk group (risk group based on ctDNA levels).

DART 2.0 (NCT05541016) is a study evaluating different de-intensification strategies using the ctDNA levels as a stratification variable. The low-risk group receives observation alone, while two other groups receive adjuvant radiation based on ctDNA levels.

## 6. Conclusions

An emerging body of evidence suggests an evolving role for measuring ctDNA as an adjunct to standard care pathways for HPV-OPC. In the diagnostic setting, ctDNA (in particular, detected by ddPCR) has a sensitivity in the 90–98% range and a specificity approaching 100%. On occasion, squamous cell carcinoma is diagnosed but with insufficient tissue for p16 staining (often on fine-needle aspiration samples). In such a situation, plasma ctDNA may help differentiate between HPV-negative and HPV-positive disease in lieu of repeating the biopsy. Moreover, ctDNA levels before treatment and in the initial phase of treatment have been shown to predict therapeutic responses. Furthermore, the detection of ctDNA after treatment has been shown to precede biopsy-proven recurrences by up to 4 months. Finally, ctDNA may have a role in cost-effectiveness. In a review, Haring et al. calculated the 2021 CMS costs for 10-year surveillance in OPSCC to be $17,381.68 [18]. Kowalchuk et al. reported the findings of a cross-sectional study comparing the cost and effectiveness of three surveillance strategies in post-treatment settings [49]. The NCCN recommendation for a posttreatment PET/CT following treatment, clinical examination, and nasopharyngoscopy every 3 months for the first 2 years was found to have a median cost of $12,780 ($11,765–$15,331), whereas replacing surveillance imaging with ctDNA every 3 months was found to have a median cost of $8541 ($8474–$10,620). The actual cost-effectiveness of ctDNA for HPV-driven tumors is difficult to estimate due to limited availability, the absence of FDA approval, and complex insurance coverage. Additionally, the cost and equipment availability will be an issue for low-resource settings. However, ongoing wide adoption will reduce the cost of the procedure.

Ongoing clinical trials are also exploring the role of ctDNA in selecting patients for de-intensification strategies. As new data emerge, the possibility of better care pathways heralds improved outcomes for patients with HPV-OPC.

## Figures and Tables

**Table 1:** Literature-reported sensitivity and specificity rates for ctHPV DNA detection.

Author	ctDNA Diagnostic	Target	Sensitivity	Specificity
Ahn et al. [8]	qPCR	HPV16 E6 and E7	67% (*n* = 93)	100% (*n* = 9)
Siravegna et al. [28]	ddPCR	HPV E7 (multiple strains)	98% (*n* = 70)	99% (*n* = 70)
Mattox et al. [22]	ddPCR	HPV16 E6	8% (*n* = 66)	
	qPCR	HPV16 E6	2%	
	NGS	HPV16 E6	75%	
Veyer et al. [29]	ddPCR	HPV E6	71% (*n* = 66)	
Tanaka et al. [30]	ddPCR	HPV E6 and E7	67% (*n* = 93)	97% (*n* = 32)
Hanna et al. [31]	ddPCR	HPV E7	76% (*n* = 21)	
Chera et al. [32]	ddPCR	HPV16 E7 and additional strains	89% (*n* = 103)	97% (*n* = 115)
Cao et al. [33]	Conventional PCR	HPV E6 and E7	65% (*n* = 40)	
Wang et al. [34]	ddPCR	HPV16 and 18 E7	91% (*n* = 22)	
Rettig et al. [25]	ddPCR	Unspecified HPV sequences and mutated oncogenes	43% (*n* = 7)	100% (*n* = 100)
Leung et al. [35]	HPVseq	E6 and E7 and full-length HPV strains including 16 and 18	100% (*n* = 17)	
Tewari et al. [36]	Conventional PCR	HPV DNA		100% (*n* = 407)

**Table 2:** Studies investigating ctHPV DNA as a cancer surveillance tool after definitive treatment.

Author/Trial Name	Number of Cases	Study Design	Detection Method	Definitive Treatment
Cao et al. [33]	14	SI, RA	qPCR	CRT
Routman et al. [43]	32	SI, RA	qPCR	Surgery
Ahn et al. [8]	93	SI, RA	qPCR	CRT/surgery
Berger et al. [44]	1076	MI, RA	ddPCR	CRT/surgery
Agrawal et al. [45]	135	SI, RA	qPCR	CRT/surgery
Veyer et al. [29]	66	SI, RA	ddPCR	CRT/surgery
Haring [46]	34	SI, PA	ddPCR	CRT/Surgery
Chera [47]	115	SI, PA	qPCR	CRT
Rutkowski [48]	66	SI, RA	qPCR	CRT

SI—single institutional study; MI—multi-institutional study; RA—retrospective analysis; PA—prospective analysis.

## Data Availability

Not applicable.

## References

[1] Damgacioglu H., Sonawane K., Zhu Y., Li R., Balasubramanian B.A., Lairson D.R., Giuliano A.R., Deshmukh A.A. (2022). Oropharyngeal Cancer Incidence and Mortality Trends in All 50 States in the US, 2001–2017. JAMA Otolaryngol.-Head Neck Surg..

[2] Taberna M., Mena M., Pavon M.A., Alemany L., Gillison M.L., Mesia R. (2017). Human papillomavirus-related oropharyngeal cancer. Ann. Oncol..

[3] Mehanna H., Rischin D., Wong S.J., Gregoire V., Ferris R., Waldron J., Le Q.T., Forster M., Gillison M., Laskar S. (2020). De-Escalation After DE-ESCALATE and RTOG 1016: A Head and Neck Cancer InterGroup Framework for Future De-Escalation Studies. J. Clin. Oncol..

[4] Lee N., Schoder H., Beattie B., Lanning R., Riaz N., McBride S., Katabi N., Li D.A., Yarusi B., Chan S.S.E. (2016). Strategy of Using Intratreatment Hypoxia Imaging to Selectively and Safely Guide Radiation Dose De-escalation Concurrent With Chemotherapy for Locoregionally Advanced Human Papillomavirus-Related Oropharyngeal Carcinoma. Int. J. Radiat. Oncol. Biol. Phys..

[5] Mirghani H., Amen F., Blanchard P., Moreau F., Guigay J., Hartl D.M., St Guily J.L. (2015). Treatment de-escalation in HPV-positive oropharyngeal carcinoma: Ongoing trials, critical issues and perspectives. Int. J. Cancer.

[6] Chan K.C.A., Woo J.K.S., King A., Zee B.C.Y., Lam W.K.J., Chan S.L., Chu S.W.I., Mak C., Tse I.O.L., Leung S.Y.M. (2017). Analysis of Plasma Epstein–Barr Virus DNA to Screen for Nasopharyngeal Cancer. N. Engl. J. Med..

[7] Wang Y., Zhao C., Chang L., Jia R., Liu R., Zhang Y., Gao X., Li J., Chen R., Xia X. (2019). Circulating tumor DNA analyses predict progressive disease and indicate trastuzumab-resistant mechanism in advanced gastric cancer. EBioMedicine.

[8] Ahn S.M., Chan J.Y., Zhang Z., Wang H., Khan Z., Bishop J.A., Westra W., Koch W.M., Califano J.A. (2014). Saliva and plasma quantitative polymerase chain reaction-based detection and surveillance of human papillomavirus-related head and neck cancer. JAMA Otolaryngol.-Head Neck Surg..

[9] Lin J.-C., Wang W.-Y., Chen K.Y., Wei Y.-H., Liang W.-M., Jan J.-S., Jiang R.-S. (2004). Quantification of Plasma Epstein–Barr Virus DNA in Patients with Advanced Nasopharyngeal Carcinoma. N. Engl. J. Med..

[10] Jeannot E., Becette V., Campitelli M., Calméjane M.A., Lappartient E., Ruff E., Saada S., Holmes A., Bellet D., Sastre-Garau X. (2016). Circulating human papillomavirus DNA detected using droplet digital PCR in the serum of patients diagnosed with early stage human papillomavirus-associated invasive carcinoma. J. Pathol. Clin. Res..

[11] Kustanovich A., Schwartz R., Peretz T., Grinshpun A. (2019). Life and death of circulating cell-free DNA. Cancer Biol..

[12] Kato S., Schwaederlé M.C., Fanta P.T., Okamura R., Leichman L., Lippman S.M., Lanman R.B., Raymond V.M., Talasaz A., Kurzrock R. (2019). Genomic Assessment of Blood-Derived Circulating Tumor DNA in Patients With Colorectal Cancers: Correlation With Tissue Sequencing, Therapeutic Response, and Survival. JCO Precis Oncol..

[13] Yamamoto Y., Uemura M., Nakano K., Hayashi Y., Wang C., Ishizuya Y., Kinouchi T., Hayashi T., Matsuzaki K., Jingushi K. (2018). Increased level and fragmentation of plasma circulating cell-free DNA are diagnostic and prognostic markers for renal cell carcinoma. Oncotarget.

[14] Łochowska B.A., Nowak D., Białasiewicz P. (2019). Cell-free tumour DNA as a diagnostic and prognostic biomarker in non-small cell lung carcinoma. Adv. Respir. Med..

[15] Mithraprabhu S., Morley R., Khong T., Kalff A., Bergin K., Hocking J., Savvidou I., Bowen K.M., Ramachandran M., Choi K. (2019). Monitoring tumour burden and therapeutic response through analysis of circulating tumour DNA and extracellular RNA in multiple myeloma patients. Leukemia.

[16] Hamfjord J., Guren T.K., Dajani O., Johansen J.S., Glimelius B., Sorbye H., Pfeiffer P., Lingjærde O.C., Tveit K.M., Kure E.H. (2019). Total circulating cell-free DNA as a prognostic biomarker in metastatic colorectal cancer before first-line oxaliplatin-based chemotherapy. Ann. Oncol..

[17] Choudhury J.H., Ghosh S.K. (2015). Promoter Hypermethylation Profiling Identifies Subtypes of Head and Neck Cancer with Distinct Viral, Environmental, Genetic and Survival Characteristics. PLoS ONE.

[18] Haring C.T., Dermody S.M., Yalamanchi P., Kang S.Y., Old M.O., Brenner J.C., Spector M.E., Rocco J.W. (2022). The future of circulating tumor DNA as a biomarker in HPV related oropharyngeal squamous cell carcinoma. Oral Oncol..

[19] Pang J., Nguyen N., Luebeck J., Ball L., Finegersh A., Ren S., Nakagawa T., Flagg M., Sadat S., Mischel P.S. (2021). Extrachromosomal DNA in HPV-Mediated Oropharyngeal Cancer Drives Diverse Oncogene Transcription. Clin. Cancer Res..

[20] Ren S., Gaykalova D.A., Guo T., Favorov A.V., Fertig E.J., Tamayo P., Callejas-Valera J.L., Allevato M., Gilardi M., Santos J. (2020). HPV E2, E4, E5 drive alternative carcinogenic pathways in HPV positive cancers. Oncogene.

[21] Johnson N.W., Salinas Montalvo A.M., McMillan N.A.J. (2022). Plasma Circulating Tumor HPV DNA and HPV-Related Oropharynx Cancer-A Caution. JAMA Otolaryngol.-Head Neck Surg..

[22] Mattox A.K., D’Souza G., Khan Z., Allen H., Henson S., Seiwert T.Y., Koch W., Pardoll D.M., Fakhry C. (2022). Comparison of next generation sequencing, droplet digital PCR, and quantitative real-time PCR for the earlier detection and quantification of HPV in HPV-positive oropharyngeal cancer. Oral Oncol..

[23] O’Sullivan B., Huang S.H., Su J., Garden A.S., Sturgis E.M., Dahlstrom K., Lee N., Riaz N., Pei X., Koyfman S.A. (2016). Development and validation of a staging system for HPV-related oropharyngeal cancer by the International Collaboration on Oropharyngeal cancer Network for Staging (ICON-S): A multicentre cohort study. Lancet Oncol..

[24] Geltzeiler M., Bertolet M., Albergotti W., Gleysteen J., Olson B., Persky M., Gross N., Li R., Andersen P., Kim S. (2018). Staging HPV-related oropharyngeal cancer: Validation of AJCC-8 in a surgical cohort. Oral Oncol..

[25] Rettig E.M., Faden D.L., Sandhu S., Wong K., Faquin W.C., Warinner C., Stephens P., Kumar S., Kuperwasser C., Richmon J.D. (2022). Detection of circulating tumor human papillomavirus DNA before diagnosis of HPV-positive head and neck cancer. Int. J. Cancer.

[26] De Ferreira C.C., Dufloth R., de Carvalho A.C., Reis R.M., Santana I., Carvalho R.S., Gama R.R. (2021). Correlation of p16 immunohistochemistry with clinical and epidemiological features in oropharyngeal squamous-cell carcinoma. PLoS ONE.

[27] Jeannot E., Latouche A., Bonneau C., Calméjane M.A., Beaufort C., Ruigrok-Ritstier K., Bataillon G., Larbi Chérif L., Dupain C., Lecerf C. (2021). Circulating HPV DNA as a Marker for Early Detection of Relapse in Patients with Cervical Cancer. Clin. Cancer Res..

[28] Siravegna G., O’Boyle C.J., Varmeh S., Queenan N., Michel A., Stein J., Thierauf J., Sadow P.M., Faquin W.C., Perry S.K. (2022). Cell-Free HPV DNA Provides an Accurate and Rapid Diagnosis of HPV-Associated Head and Neck Cancer. Clin. Cancer Res..

[29] Veyer D., Wack M., Mandavit M., Garrigou S., Hans S., Bonfils P., Tartour E., Bélec L., Wang-Renault S.F., Laurent-Puig P. (2020). HPV circulating tumoral DNA quantification by droplet-based digital PCR: A promising predictive and prognostic biomarker for HPV-associated oropharyngeal cancers. Int. J. Cancer.

[30] Tanaka H., Suzuki M., Takemoto N., Fukusumi T., Eguchi H., Takai E., Kanai H., Tatsumi M., Horie M., Takenaka Y. (2022). Performance of oral HPV DNA, oral HPV mRNA and circulating tumor HPV DNA in the detection of HPV-related oropharyngeal cancer and cancer of unknown primary. Int. J. Cancer.

[31] Hanna G.J., Lau C.J., Mahmood U., Supplee J.G., Mogili A.R., Haddad R.I., Jänne P.A., Paweletz C.P. (2019). Salivary HPV DNA informs locoregional disease status in advanced HPV-associated oropharyngeal cancer. Oral Oncol..

[32] Chera B.S., Kumar S., Beaty B.T., Marron D., Jefferys S., Green R., Goldman E.C., Amdur R., Sheets N., Dagan R. (2019). Rapid Clearance Profile of Plasma Circulating Tumor HPV Type 16 DNA during Chemoradiotherapy Correlates with Disease Control in HPV-Associated Oropharyngeal Cancer. Clin. Cancer Res..

[33] Cao H., Banh A., Kwok S., Shi X., Wu S., Krakow T., Khong B., Bavan B., Bala R., Pinsky B.A. (2012). Quantitation of human papillomavirus DNA in plasma of oropharyngeal carcinoma patients. Int. J. Radiat. Oncol. Biol. Phys..

[34] Wang Y., Springer S., Mulvey C.L., Silliman N., Schaefer J., Sausen M., James N., Rettig E.M., Guo T., Pickering C.R. (2015). Detection of somatic mutations and HPV in the saliva and plasma of patients with head and neck squamous cell carcinomas. Sci. Transl. Med..

[35] Leung E., Han K., Zou J., Zhao Z., Zheng Y., Wang T.T., Rostami A., Siu L.L., Pugh T.J., Bratman S.V. (2021). HPV Sequencing Facilitates Ultrasensitive Detection of HPV Circulating Tumor DNA. Clin. Cancer Res..

[36] Tewari S.R., D’Souza G., Troy T., Wright H., Struijk L., Waterboer T., Fakhry C. (2022). Association of Plasma Circulating Tumor HPV DNA With HPV-Related Oropharynx Cancer. JAMA Otolaryngol. Head Neck Surg..

[37] Asheer J., Jensen J.S., Grønhøj C., Jakobsen K.K., Buchwald C.v. (2020). Rate of locoregional recurrence among patients with oropharyngeal squamous cell carcinoma with known HPV status: A systematic review. Acta Oncol..

[38] Nguyen-Tan P.F., Zhang Q., Ang K.K., Weber R.S., Rosenthal D.I., Soulieres D., Kim H., Silverman C., Raben A., Galloway T.J. (2014). Randomized phase III trial to test accelerated versus standard fractionation in combination with concurrent cisplatin for head and neck carcinomas in the Radiation Therapy Oncology Group 0129 trial: Long-term report of efficacy and toxicity. J. Clin. Oncol..

[39] Ang K.K., Harris J., Wheeler R., Weber R., Rosenthal D.I., Nguyen-Tân P.F., Westra W.H., Chung C.H., Jordan R.C., Lu C. (2010). Human papillomavirus and survival of patients with oropharyngeal cancer. N. Engl. J. Med..

[40] Cao Y., Haring C.T., Brummel C., Bhambhani C., Aryal M., Lee C., Neal M.H., Bhangale A., Gu W.J., Casper K. (2022). Early HPV ctDNA Kinetics and Imaging Biomarkers Predict Therapeutic Response in p16+Oropharyngeal Squamous Cell Carcinoma. Clin. Cancer Res..

[41] Dahlstrom K.R., Li G., Hussey C.S., Vo J.T., Wei Q., Zhao C., Sturgis E.M. (2015). Circulating human papillomavirus DNA as a marker for disease extent and recurrence among patients with oropharyngeal cancer. Cancer.

[42] Guo T., Kang S.Y., Cohen E.E.W. (2022). Current perspectives on recurrent HPV-mediated oropharyngeal cancer. Front. Oncol..

[43] Routman D.M., Kumar S., Chera B.S., Jethwa K.R., Van Abel K.M., Frechette K., DeWees T., Golafshar M., Garcia J.J., Price D.L. (2022). Detectable Postoperative Circulating Tumor Human Papillomavirus DNA and Association with Recurrence in Patients With HPV-Associated Oropharyngeal Squamous Cell Carcinoma. Int. J. Radiat. Oncol. Biol. Phys..

[44] Berger B.M., Hanna G.J., Posner M.R., Genden E.M., Lautersztain J., Naber S.P., Del Vecchio Fitz C., Kuperwasser C. (2022). Detection of Occult Recurrence Using Circulating Tumor Tissue Modified Viral HPV DNA among Patients Treated for HPV-Driven Oropharyngeal Carcinoma. Clin. Cancer Res..

[45] Agrawal Y., Koch W.M., Xiao W., Westra W.H., Trivett A.L., Symer D.E., Gillison M.L. (2008). Oral Human Papillomavirus Infection Before and After Treatment for Human Papillomavirus 16–Positive and Human Papillomavirus 16–Negative Head and Neck Squamous Cell Carcinoma. Clin. Cancer Res..

[46] Haring C.T., Bhambhani C., Brummel C., Jewell B., Bellile E., Heft Neal M.E., Sandford E., Spengler R.M., Bhangale A., Spector M.E. (2021). Human papilloma virus circulating tumor DNA assay predicts treatment response in recurrent/metastatic head and neck squamous cell carcinoma. Oncotarget.

[47] Chera B.S., Kumar S., Shen C., Amdur R., Dagan R., Green R., Goldman E., Weiss J., Grilley-Olson J., Patel S. (2020). Plasma Circulating Tumor HPV DNA for the Surveillance of Cancer Recurrence in HPV-Associated Oropharyngeal Cancer. J. Clin. Oncol..

[48] Rutkowski T.W., Mazurek A.M., Snietura M., Hejduk B., Jedrzejewska M., Bobek-Billewicz B., d’Amico A., Piglowski W., Wygoda A., Skladowski K. (2020). Circulating HPV16 DNA may complement imaging assessment of early treatment efficacy in patients with HPV-positive oropharyngeal cancer. J. Transl. Med..

[49] Kowalchuk R.O., Kamdem Talom B.C., Van Abel K.M., Ma D.M., Waddle M.R., Routman D.M. (2022). Estimated Cost of Circulating Tumor DNA for Posttreatment Surveillance of Human Papillomavirus–Associated Oropharyngeal Cancer. JAMA Netw. Open.

